# Dietary 25-Hydroxyvitamin D3 Supplementation Modulates Intestinal Cytokines in Young Broiler Chickens

**DOI:** 10.3389/fvets.2022.947276

**Published:** 2022-07-11

**Authors:** Gerardo A. Abascal-Ponciano, Samuel F. Leiva, Joshua J. Flees, Luis P. Avila, Jessica D. Starkey, Charles W. Starkey

**Affiliations:** Department of Poultry Science, Auburn University, Auburn, AL, United States

**Keywords:** 25-hydroxyvitamin D, cytokine, inflammatory responses, interleukin, intestine, tight junctions

## Abstract

Vitamin D signaling is important for intestinal homeostasis. An increase in vitamin D receptors in immune cells can modulate cell phenotype and cytokine secretion. Cytokines regulate both pro- (interleukin 17; IL-17) and anti-inflammatory (IL-10) responses triggered by external stimuli. Inflammation in intestinal tissues can disrupt the structure and the remodeling of epithelial tight junction complexes, thus, compromising the protective barrier. The objective of the study was to determine the impact of dietary supplementation with 25-hydroxycholecalciferol (_25_OHD_3_), a hydroxylated metabolite of vitamin D, on intestinal cytokine abundance and epithelial barrier integrity over time in broilers. A randomized complete block design experiment was conducted to evaluate the effect of dietary _25_OHD_3_ inclusion on relative protein expression of the cytokines, IL-17 and IL-10, and tight junction proteins, Zona Occludens 1 (ZO-1), and Claudin-1 (CLD-1), in broiler chicken duodenum and ileum from 3 to 21 days post-hatch. On day 0, male chicks (*n* = 168) were randomly assigned to raised floor pens. Experimental corn–soybean meal-based treatments were as follows: (1) a common starter diet containing 5,000 IU of D_3_ per kg of feed (VITD_3_) and (2) a common starter diet containing 2,240 IU of D_3_ + 2,760 IU of _25_OHD_3_ per kg of feed (_25_OHD_3_) fed from days 0 to 21. On days 3, 6, 9, 12, 15, 18, and 21, 12 birds per treatment were euthanized to collect tissue samples for quantitative, multiplex, and fluorescent Western blot analysis. Target proteins were quantified using Image Quant TL 8.1 and expressed relative to total protein. Feeding _25_OHD_3_ post-hatch decreased ileal IL-10 (anti-inflammatory) protein expression in 21-day-old broilers compared with VITD_3_ only (*P* = 0.0190). Broilers fed only VITD_3_ post-hatch had greater IL-17 (pro-inflammatory) protein expression in the ileum at 18 and 21 days-of-age (*P* = 0.0412) than those that fed _25_OHD_3_. Dietary inclusion of _25_OHD_3_ lowered the abundance of key inflammatory cytokines in the ileum of young broilers.

## Introduction

Poultry enteric challenges are commonly caused by bacteria or parasites. These challenges cost the United States poultry industry over US$1.6 billion in losses every year (1, 2). Pathogenic and non-pathogenic bacteria are common inhabitants of a broiler gastrointestinal tract. The proliferation of pathogenic, toxin-secreting strains of gut bacteria promotes intestinal upsets in broiler chickens. To mitigate these challenges, immunological responses in the gastrointestinal tract take place near the epithelial cell wall. Upon pathogen recognition by toll-like receptors, innate immune cells are activated and initiate a signaling cascade, recruiting phagocytes to the site of infection. Inflammation is a by-product of innate immune responses that can decrease feed intake (3, 4) and lead to lower availability and utilization of nutrients (5). In addition, inflammatory responses can distort intestinal integrity by modulating epithelial tight junction complexes (6, 7). The cost of inflammation stems from nutrient mobilization toward immune mechanisms and loss of tissue function from collateral damage caused by such mechanisms (8).

Immune cells, such as macrophages and lymphocytes, secrete signaling molecules to regulate inflammatory actions. Cytokines and other regulatory peptides modulate proliferation or inhibition of different cell types related to inflammation. Pro-inflammatory cytokines, such as IL-17, are associated with inflammation and the recruitment of phagocytes to the infection area. Comparably, IL-10 is an anti-inflammatory cytokine that acts on target cells to reduce or inhibit the production of pro-inflammatory cytokines. Inflammation is a component of necessary immune reactions that clearly modulate epithelial barrier integrity (9). Antibiotic growth-promoters (AGPs) are used in feed to inhibit populations of *Clostridium perfringens* and pathogens (10). Regulating bacterial populations in the gut can reduce immunological responses and consequently inflammation.

Concerns over AGP use in feed have encouraged the evaluation of other nutritional strategies to support immune function and disease prevention in poultry. The microbial effects of several probiotics and other feed additives have been assessed in poultry gastrointestinal tracts (11, 12). Lu et al. (13) reported similar inflammatory cytokine IL-6 modulation by AGP and plant or yeast-based antibiotic alternatives in young broilers orally vaccinated with *Eimeria* species. Common feed ingredients, such as minerals and vitamins, are necessary for optimal health and performance. Dietary supplementation of vitamins A, E, and D has been shown to play crucial roles in avian immune modulation (14–16), and their inclusion in poultry diets supports humoral and immune responses.

Vitamin D is a fat-soluble vitamin known for its role in calcium and phosphorus homeostasis and bone mineralization. Different metabolites of vitamin D can be included in production animal diets. Dietary vitamin D is supplied in poultry diets as vitamin D_3_ or as a combination of D_3_ and 25-hydroxyvitamin D_3_ (_25_OHD3), a hydroxylated form of vitamin D_3_ and major circulating vitamin D metabolite (17). The presence of the vitamin D receptor in cells unrelated to mineral metabolism suggests that vitamin D plays an important role in more tissues (18). Vitamin D and its metabolites have been studied in other areas, such as muscle development and immunity. Dietary addition of _25_OHD_3_ has resulted in increased broiler performance relative to vitamin D_3_ supplementation only (19). Both pro- and anti-inflammatory immunomodulatory effects have been reported in the intestine and immune organs of immune-challenged birds fed _25_OHD_3_ compared to vitamin D_3_ only diets (20, 21). Furthermore, dietary inclusion of _25_OHD_3_ modulates gene expression of intestinal tight junctions in layer hens under stress conditions (22). Immunomodulatory effects of _25_OHD_3_ in intestinal tissues of broiler chickens could regulate local inflammation and support epithelium integrity. This study was designed to determine the impact of dietary supplementation with _25_OHD_3_ on intestinal cytokine abundance and epithelial barrier integrity over time in broilers.

## Materials and Methods

The Auburn University Institutional Animal Care and Use Committee approved the use of live birds in this experimental protocol (PRN 2017-3212).

### Bird Husbandry

Broiler chicks were raised as described by Leiva et al. (23). In brief, day-old, male, unvaccinated Yield Plus × Ross 708 broiler chicks were obtained from the University of Georgia at Athens (*n* = 168). On the day of placement, chicks were identified with a wing tag and randomly allotted in groups of four to raised floor pens (0.05 m^2^/bird) located in a solid-sided, temperature-controlled, and dehumidified research facility. Each pen was bedded with new pine shavings, contained individual metal feeders, and 2 nipple waterers. During days 1 through 7, a supplemental plastic feeder was included in each pen to facilitate feed consumption. On day 7, broilers were reallocated to individual pens (*n* = 168; *n* = 84 pens/treatment) with their correspondent dietary treatment (*n* = 1 bird/pen; 0.20 m^2^/pen). The ambient temperature was set to 32°C at placement and gradually reduced to 21°C by day 21 to maintain bird comfort. Birds were reared with a photoperiod of 23 h at 30 lux of light intensity during the first 7 days of age, followed by an 18 h light period at 10 lux from days 8 to 21. Broilers were fed 1 of 2 corn and soybean-meal–based treatment diets formulated to contain 5,000 IU of D_3_ per kg of feed (VITD_3_), or 2,240 IU of D_3_ (Rovimix D_3_ 500; DSM Nutritional Products Inc., Parsippany, NJ) + 2,760 IU of _25_OHD_3_ (69 μg Rovimix Hy-D; DSM Nutritional Products Inc.) per kg of feed (_25_OHD_3_). Feed was provided as a starter crumble diet throughout the experiment (day 1 to 21), and feed and water were offered *ad libitum*. Diets were analyzed by DSM Nutritional Products TMAS laboratory (Beldivere, NJ). Association of Official Agricultural Chemists (AOAC) official method 2011.12 was used to measure vitamin D_3_ content (Table 1). The concentration of _25_OHD_3_ in the feed was measured using HPLC and mass spectroscopy (Table 1).

**Table 1:** Composition of broiler diets.

**Ingredients, %**	**Broiler diet^a^**	
	**D3**	**25OHD3**
Ground corn	50.96	50.96
Soybean meal	38.50	38.50
Dried distiller grains	3.00	3.00
Soybean oil^b^	2.50	2.50
Dicalcium phosphate	1.80	1.80
Limestone (fine)	1.50	1.50
Salt	0.40	0.40
DL-Methionine	0.35	0.35
L-Lysine	0.15	0.15
L-Threonine	0.04	0.04
Choline chloride 60%	0.10	0.10
Trace mineral premix^c^	0.10	0.10
No-D_3_ Vitamin premix^d^	0.05	0.05
D_3_ premixed hand-add^e^	0.05	-
_25_OHD_3_ hand-add^f^	-	0.50
Amprol (Coccidiostat)	0.05	0.05
Total	100.00	100.00
**Calculated nutrients**
Vitamin D3, IU per kg of diet	5,000.00	2,240.00
25-Hydroxycholecalciferol, IU per kg	-	2,760.00
Crude protein, %	23.40	23.40
Ca, %	1.00	1.00
Available P, %	0.40	0.40
ME, kcal per kg of diet	2,940.00	2,940.00
Digestible lysine, %	1.20	1.20
Digestible methionine, %	0.60	0.60
**Analyzed nutrients**
Vitamin D3, IU per kg of diet	4,390.00	2,144.00
25-Hydroxycholecalciferol, IU per kg	-	1,978.00

a *Starter (crumble): days 0 to 21*.

b *>Sprayed 1.5% post-pellet*.

c *>Supplied per kg of diet: Mn, 109 mg as Mn sulfate; Zn, 90 mg as Zn sulfate; Fe, 27 mg as ferrous sulfate; Cu, 7 mg as basic Cu chloride; I, 1.3 mg as ethylenediamine hydroiodide; and Se, 0.3 mg as sodium selenite*.

d *>Supplied per kg of diet: vitamin A, 12,474 IU; vitamin E, 100 mg; vitamin K, 4 mg; thiamine, 3 mg; riboflavin, 8 mg; pyridoxin, 4 mg; cobalamin, 0.03 mg; niacin, 60 mg; pantothenic acid, 15 mg; folic acid, 2.5 mg; and biotin, 0.4 mg*.

e *>Supplied per kg of diet: 5,000 IU of vitamin D3*.

f *>Supplied per kg of diet: 69 μg of 25-hydroxycholecalciferol equivalent to 2,760 IU*.

### Sample Collection

On days 3, 6, 9, 12, 15, 18, and 21 post-hatch, 12 birds per treatment from 12 randomly selected blocks were euthanized by cervical dislocation. Duodenum tissue samples were collected just distal to the duodenal loop to prevent the inclusion of pancreatic tissue. Ileum tissue samples were collected 4 cm proximal to the cecal tonsil attachment. Following excision of tissues, samples were immediately flushed with tris buffered saline (TBS; pH 7.4; Thermo Fisher Scientific, Fair Lawn, NJ) to remove intestinal contents, flash-frozen in liquid nitrogen, and stored at −80°C prior to analysis for protein expression by quantitative, fluorescent Western Blotting as described later.

### Protein Extraction

Duodenum and ileum tissue samples (≈100 mg) were placed in ice-cold T-PER lysis buffer (Cat. No. 78510; Thermo Fisher Scientific) supplemented with a 2X final concentration of Halt protease and phosphatase inhibitor cocktail (Cat. No. 78441; Thermo Fisher Scientific). Samples were homogenized using a Qiagen TissueLyser II (Cat. No. 85300; Qiagen, Germantown, MD, United States) two times at 30 Hz for 2 min. Following homogenization, samples were centrifuged at 12,000 × *g* for 10 min. Supernatants were removed carefully and protein concentrations were determined using a Pierce BCA Protein Assay Kit (Cat. No. 23225; Thermo Fisher Scientific) with a NanoDrop One spectrophotometer (ND-ONEC-W; Thermo Fisher Scientific). The stock sample was divided into aliquots and stored at−80°C until analysis.

### Primary and Secondary Antibodies

Primary antibodies used were as follows: mouse monoclonal IgG2b anti-IL-10 (1:1,000 dilution; Cat. No. SC-365858; Santa Cruz Biotechnology; Santa Cruz, CA, USA); rabbit polyclonal IgG anti-Chicken IL-17 (1:2,000 dilution; Cat. No. LS C294849; Lifespan Biosciences; Seattle, WA, USA); mouse monoclonal IgG2b anti-claudin-1 (1:300 dilution; Cat. No. SC-166338; Santa Cruz Biotechnology; Santa Cruz, CA, USA); and rabbit polyclonal IgG anti-Rat TJP1/ZO-1 (1:1,500 dilution; Cat. No. LS C145545; Lifespan Biosciences; Seattle, WA, USA). Primary antibodies were detected using the following secondary antibodies (1:10,000 dilution; Invitrogen, Thermo Fisher Scientific Inc.; Waltham, MA, USA): Alexa-Fluor 488 conjugated goat anti-mouse IgG1 (Cat. No. A-11008) and Alexa-Fluor 555 Plus conjugated goat anti-mouse IgG (Cat. No. A-32727).

### Western Blotting

Samples at 30 μg of total protein were mixed with T-PER lysis buffer to achieve an 8-μl final volume. Samples used to analyze Claudin-1 expression utilized 60 μg of total protein. Samples were then mixed with 1 μl of Cy5 dye from the Amersham QuickStain Protein Labeling Kit (Cat. No. RPN4000; GE Healthcare, Chicago, IL, USA) for a total protein stain. Samples were incubated at room temperature with no light for 30 min using an Amersham QuickStain Protein Labeling Kit. Subsequently, 4X Protein Sample Loading Buffer (Cat. No. 928-40004; LI-COR Biosciences; Lincoln, NE, USA) and β-mercaptoethanol were added to each sample to achieve a final concentration of 1X sample buffer and 10 mM β-mercaptoethanol. Samples were vortexed and then heated in a water bath at 95°C for 3 min. Samples were loaded onto 24-well, 4 to 20% gradient Criterion TGX precast midi gels (Cat. No. 5671095; Bio-Rad, Hercules, CA, United States) with Amersham ECL Plex Fluorescent Rainbow Markers (Cat. No. RPN851E; GE Healthcare) added to first and last lanes of each gel as a marker of molecular weights. Gels were electrophoresed at 85 V for 15 min and then 125 V for 60 min (until the dye front reached the bottom of the gel) in a Criterion Electrophoresis Midi Vertical Cell (Cat. No. 1656001; Bio-Rad). After electrophoresis, gels were transferred to low-fluorescent polyvinylidene fluoride (PVDF) membranes from a Trans-Blot Turbo RTA Midi LF PVDF Transfer Kit (Cat. No. 1704275; Bio-Rad) using a Trans-Blot Turbo Transfer System (Cat. No. 1704150; Bio-Rad). Membranes were then blocked for 1 h at room temperature using Intercept (TBS) Blocking Buffer (Cat. No. P/N: 927-60001; LI-COR Biosciences). After blocking, membranes were incubated in primary antibodies: IL-10 and IL-17, Claudin-1, or ZO-1 diluted in Intercept T20 (TBS) Antibody Diluent (Cat. No. P/N:927-65001; LI-COR) overnight (~16 h) at 4°C. Following incubation in primary antibodies, membranes were washed three times for 5 min each in tris-buffered saline + 0.01% Tween 20 (TBST). Membranes were incubated in AlexaFluor Plus 555 and AlexaFluor 488 secondary antibodies diluted in Intercept T20 (TBS) Antibody Diluent at room temperature for 1 h. Membranes were then washed three times for 5 min each in TBST and allowed to air dry for 3 h in a dark room.

### Image Capture and Analysis

Dried membranes were imaged using an Amersham Imager 600 (Cat. No. 29083461; GE Healthcare) using fluorescent settings for green/Cy3 (IL-10, Claudin-1, and green fluorescent protein ladder markers), blue/Cy2 (IL-17 and ZO-1), and red/Cy5 (total protein and red fluorescent protein ladder markers) channels on automatic exposure time. Fluorescent band intensity for target proteins and total proteins were quantified using Image Quant TL 8.1 software (Cat. No. 29000737; GE Healthcare). Target proteins were measured relative to the total protein of each lane.

### Statistical Analysis

Data were subjected to one-way ANOVA using Statistical Analysis Software (SAS) version 9.4 (SAS Institute Inc., Cary, NC) generalized linear mixed model (GLIMMIX) procedure in which DIET served as a fixed effect. Bird (*n* = 168) served as an experimental unit. Proportional data were analyzed using the events/experiments syntax with a binomial distribution and both continuous and proportional data were analyzed using an R-side covariance structure. Pairwise least square mean comparisons were performed using the PDIFF option of SAS. Means were declared different when *P* ≤ 0.05.

## Results and Discussion

### Intestinal Cytokine Abundance

The effects of vitamin D on mammalian immunity are well documented (24). Poultry findings display conflicting immunomodulatory effects of _25_OHD_3_ on *in-vitro–*cultured chicken macrophage cell lines. Shojadoost et al. (25) reported a reduction in chemokine CXCL8 and pro-inflammatory interleukin IL-1β gene expression by _25_OHD3-pretreated chicken macrophages 24 h post-lipopolysaccharide (LPS) stimulation. Another research group concluded that chicken macrophages cultured in a _25_OHD3-supplemented medium increased mRNA levels of IL-1β and IL-10 after 48 h of LPS stimulation (26). Thus, modulation of pro- and anti-inflammatory cytokines by chicken immune cells can be attributed to _25_OHD_3_ treatment. Similar results are reported in live bird experiments, Morris et al. (27) noted lower IL-1β mRNA amounts in the liver of 28-day-old broilers fed _25_OHD_3_ compared with the broilers fed vitamin D_3_ only, following LPS injection. Yet, unchallenged broilers had similar gene expression regardless of dietary vitamin D form supplemented. Authors suggest an effect of _25_OHD_3_ on extrarenal activation of vitamin D by 1α-hydroxylase, known to catalyze the synthesis of vitamin D into its active form in the kidney. A dose-response to _25_OHD_3_ was observed in the immune organs of broilers fed low-nutrient levels. The spleen of broilers fed low calcium and phosphorous diets with the aforementioned recommended addition of _25_OHD_3_ (9,800 IU/kg) had a greater transcription of toll-like receptors and anti-inflammatory cytokines IL-4, IL-10, and IL-13 compared with those fed optimal levels of calcium or lower supplementation of _25_OHD_3_ (≤2,760 IU/kg) (28). But mRNA quantification in the bursa of Fabricius, an essential organ for the development of adaptive immunity, reveals an opposite response for IL-4 and IL-13. The effect of _25_OHD_3_ in broiler immune cells and signaling molecules is apparent when exposed to an immune challenge or stressor, but the response can vary among tissues.

**Figure 1:**
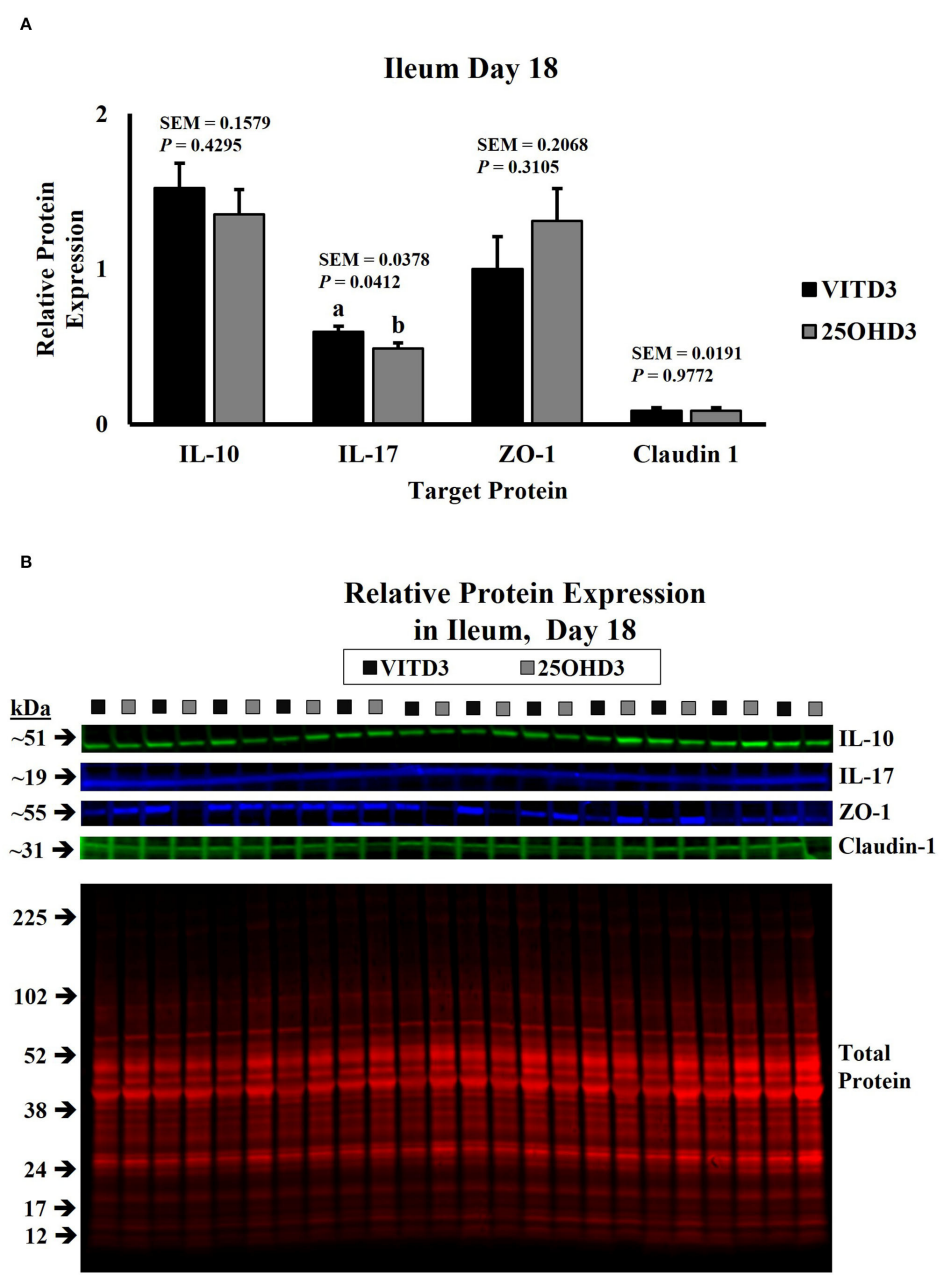
Effect of dietary 25-hydroxycholecalciferol supplementation on 18-day-old broiler chicken ileal protein abundance. In total, 24 birds were sampled on each sampling day (n = 12 birds of each treatment). Dietary treatments: VITD_3_ = 5,000 IU of vitamin D_3_ per kg of broiler chicken feed; _25_OHD_3_ = 2,760 IU of 25-hydroxycholecalciferol + 2,240 of vitamin D_3_ per kg of broiler chicken feed. Protein expression was measured using quantitative, fluorescent Western Blot relative to total protein. **(A)** IL-10, IL-17, ZO-1, and Claudin-1 protein abundance. **(B)** Day 18 representative fluorescent Western Blot. Abundance of IL-17 was grater for VITD3-fed birds. ^a,b^Bars with different superscripts differ at P ≤ 0.05.

**Figure 2:**
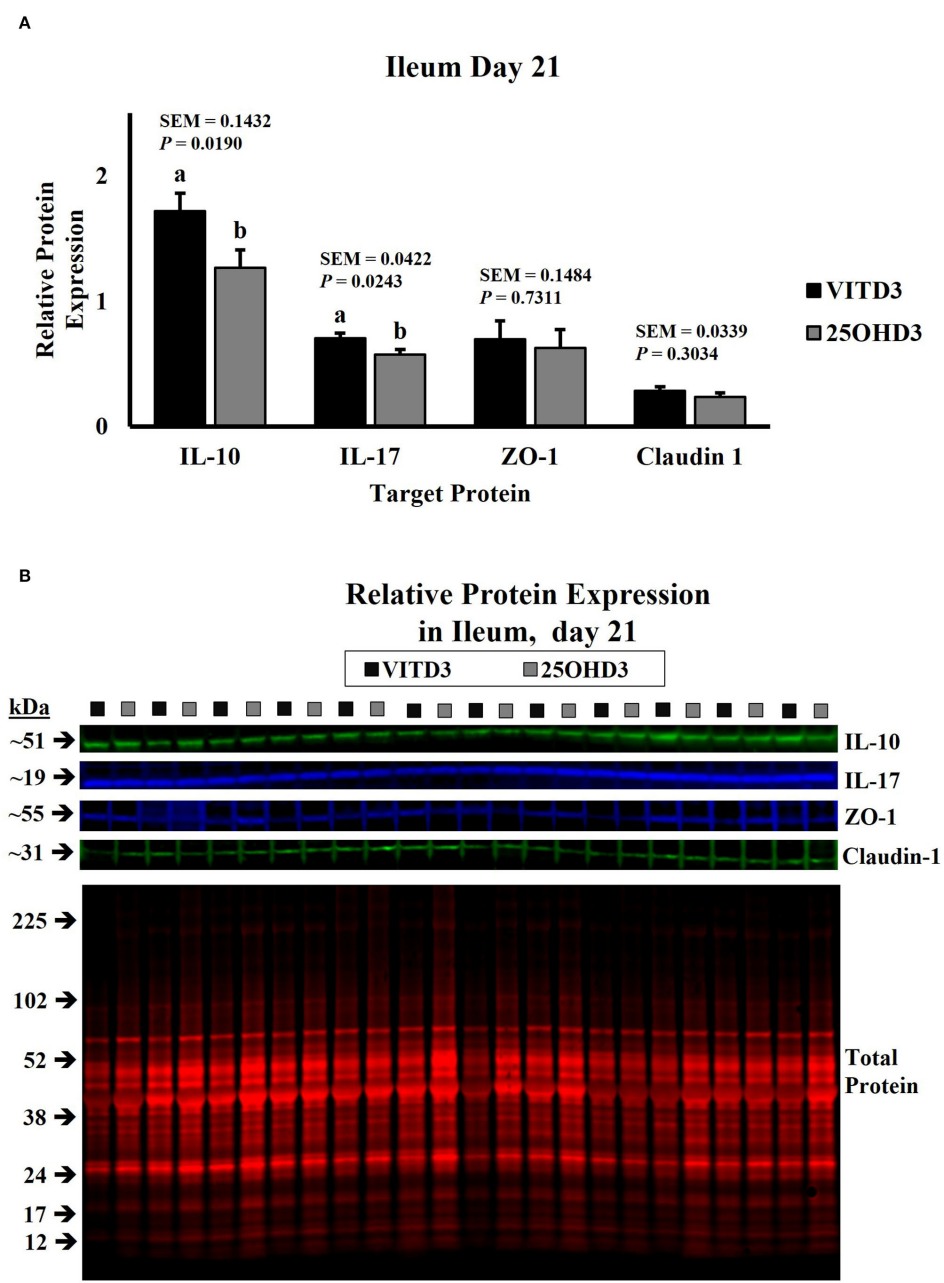
Effect of dietary 25-hydroxycholecalciferol supplementation on 21-day-old broiler chicken ileal protein abundance. In total, twenty-four birds were sampled on each sampling day (n = 12 birds of each treatment). Dietary treatments: VITD_3_ = 5,000 IU of vitamin D_3_ per kg of broiler chicken feed; _25_OHD_3_ = 2,760 IU of 25-hydroxycholecalciferol + 2,240 of vitamin D_3_ per kg of broiler chicken feed. Protein expression was measured using quantitative, fluorescent Western Blot relative to total protein. **(A)** IL-10, IL-17, ZO-1, and Claudin-1 protein abundance. **(B)** Day 21 representative fluorescent Western Blot. Abundance of IL-10 and IL-17 was grater for VITD3-fed birds. ^a,b^Bars with different superscripts differ at P ≤ 0.05.

In this study, dietary treatments did not influence the abundance of duodenal cytokines in broilers (Supplementary Figures S1–S7). However, dietary inclusion of _25_OHD_3_ in broiler diets decreased the expression of pro- and anti-inflammatory cytokines in ileal tissues of 18- and 21-day-old broilers compared with the diets containing vitamin D_3_ as the only source of vitamin D (Figures 1, 2, respectively). Similar intestinal cytokine abundance among vitamin D treatments from days 3 to 15 is supported by previous findings under unchallenged conditions (27). However, expression of pro-inflammatory IL-17 decreased in 18-day-old-broilers fed _25_OHD_3_ (0.488 vs. 0.595 relative to total protein; *P* = 0.041; Figure 1A) and this was sustained for 21-day-old-broilers (0.573 vs. 0.703 relative to total protein; *P* = 0.024; Figure 2A). Meanwhile, anti-inflammatory IL-10 abundancy also decreased in 21-day-old-broilers fed _25_OHD_3_ (1.265 vs. 1.719 relative to total protein; *P* = 0.019; Figure 2A). Literature indicates cytokine transcription differences among broilers fed different dietary vitamin D metabolites only when broilers are under a physiological stressor (27–29). Broilers used in this experiment were not intentionally exposed to immunological stress, so these differences cannot be attributed to a certain stimulus. It is reported that the addition of _25_OHD_3_ to human CD4+ T cells inhibited T helper 17 (Th17) cell differentiation (30). Th17 lymphocytes secrete pro-inflammatory IL-17 and play an important role in adaptive immunity (31, 32), hence, decreased IL-17 expression may be an indirect effect of _25_OHD_3_ on Th17 lymphocyte differentiation. In the nucleus, the ligand-binding activates the vitamin D receptor (VDR) which acts as a transcription factor (33). Recruitment of retinoid X receptors (RXR) by _1, 25_OHD_3_-bound VDR forms a heterodimer complex that binds to VDR response elements (VDREs) in promoter regions of responsive genes (34). Multiple immune-related genes in human monocytes are targeted by vitamin D (35). The interactive response of avian immune-related genes to external stimuli and different vitamin D metabolites is elusive. However, greater _25_OHD_3_ inclusions (110 vs. 25 μg/kg of feed) upregulate mRNA amounts of IL-10 and CD_4+_CD_25+_ immune cells in the cecal tonsils of turkeys challenged with *Eimeria maxima* oocyst (20), suggesting anti-inflammatory actions. Our results provide evidence of broiler cytokine modulation by different vitamin D metabolites without an intentional immune challenge.

### Intestinal Tight Junction Abundance

Epithelial cells are bound together by tight, adherent, and gap junction complexes. These proteins prevent the entry of pathogens and toxins and create selective channels to allow the passage of ions (36). Transcription of intestinal tight junctions of broilers is altered by immune challenges (37, 38) and nutrient availability (39). An experiment involving VDR knock-out mice suggests that the VDR-mediated action involves the stabilization of intestinal epithelial junction complexes (40). Furthermore, a functional VDRE is reported in the Cdx1 site of the epithelial junction Claudin-2 promoter (41). Indicating a possible direct modulation of the Claudin 2 gene by vitamin D. These claims are supported by Kühne et al. (42) who reported lower duodenal Claudin 2 abundance in VDR-knockout mice compared with the wild type mice, while all other tight junctions were similarly expressed among mice types. Evaluating the effect of vitamin D metabolites on nutrient transport across poultry intestinal epithelium has been a research focus due to the role of vitamin D in mineral metabolism (43). Studies evaluating the effect of vitamin D on broiler epithelium integrity are limited. However, Putkey and Norman (44) observed a vitamin D-induced decrease in accessibility of actin to iodination reagents in the intestinal brush border of chicks. Actin is a protein associated with the formation of tight junction complexes (45), thus, vitamin D could alter components related to gut integrity and function.

The expression of tight junction proteins in the duodenum and ileum tissue samples was similar to our experimental dietary treatments (Figures 1, 2; Supplementary Figures S1–S7). In a similar study, layer hens under stocking density stress conditions expressed lower Claudin-1 mRNA in jejunal mucosa when diets use vitamin D_3_ as the only source of vitamin D, as birds fed a combination of vitamin D_3_ and _25_OHD_3_ maintained similar Claudin-1 gene expressions under low- or high-stocking densities (22). In mammals, intestinal tight junction expression of weaned pigs increased with a porcine epidemic diarrhea virus infection challenge when 5.5 μg/kg of _25_OHD_3_ was supplemented in diets (46). Although higher _25_OHD_3_ inclusions (118 and 155.5 μg/kg of feed) did not generate this increase. Thus, modulation of intestinal tight junctions by dietary _25_OHD_3_ supplementation is subject to inclusion rates. However, _25_OHD_3_ did not influence tight junction abundance at the rate supplied in this experiment (69 μg/kg).

### Intestinal Cytokine Abundance Over Time

**Figure 3:**
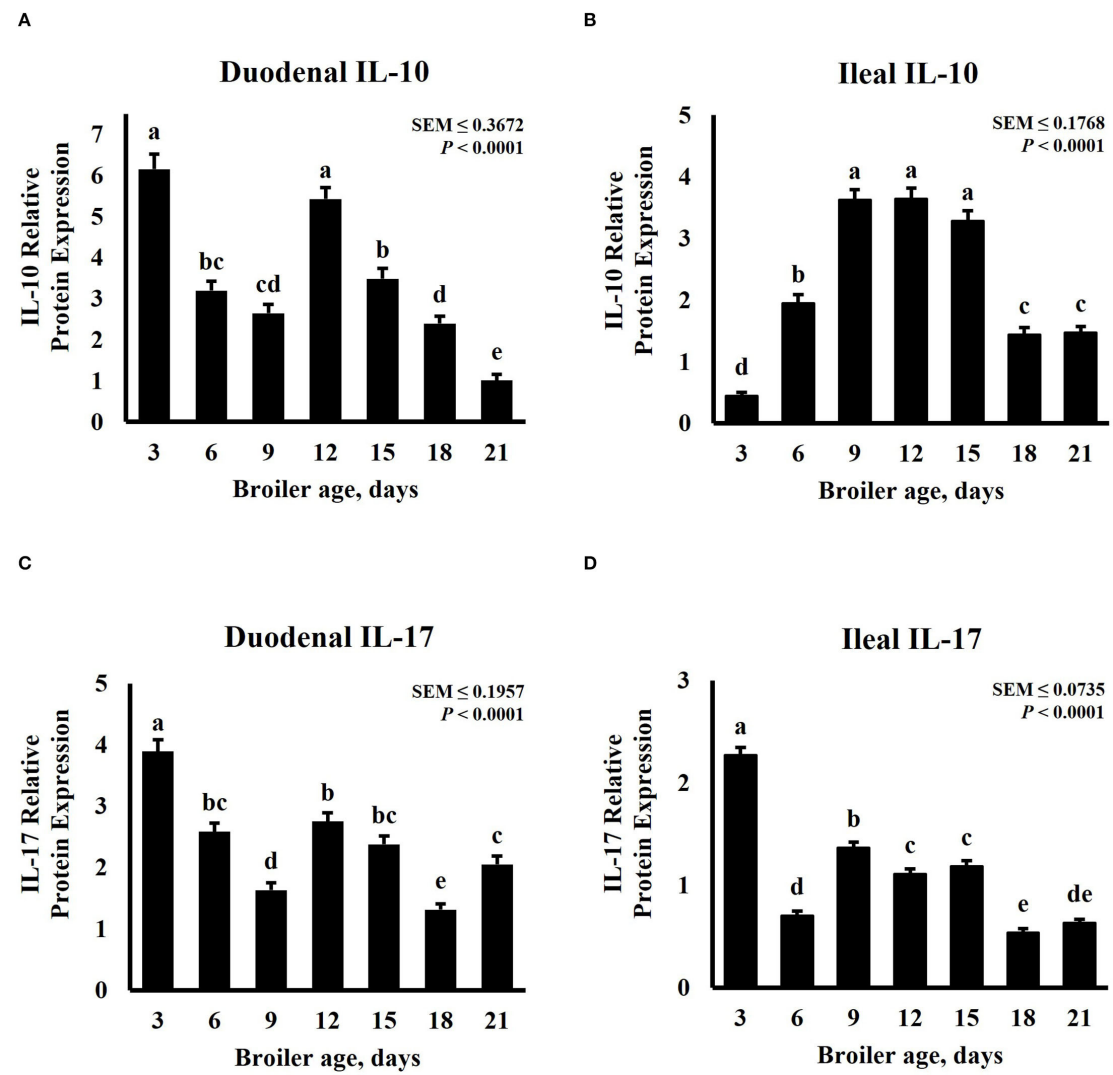
Effect of bird age on intestinal cytokine protein abundance. On each sampling day, a total of 24 birds (n = 12 birds of each treatment from 12 blocks) were selected for tissue collection. Protein expression was measured using quantitative, fluorescent Western Blot relative to total protein. **(A)** Duodenal IL-10 abundance. **(B)** Duodenal IL-17 abundance. **(C)** Ileal IL-10 abundance. **(D)** Ileal IL-17 abundance. ^a−e^Bars with different superscripts differ at P ≤ 0.05.

Intestinal cytokine modulation is affected by bird age. Xu et al. (47) observed a linear increase of anti-inflammatory cytokines genes from days 0 to 21 in the ileum of pigeon squabs, but a linear decrease for pro-inflammatory cytokines. In the broilers, an increase in mRNA expression of IL-1β and IL-2, a decrease in IL-8, and similar levels for IL-17 were detected in the jejunum of broilers from day 22 to day 28 post-hatch (48). A study involving 3 sample-collection timepoints noted a quadratic trend in IL-1β and IL-10 mRNA expression in the ceca of broilers sampled in weeks 1, 3, and 6 (49). Expression patterns of inflammatory cytokines in the duodenum and ileum for this study are presented in Figure 3. The relative abundance of both IL-10 and IL-17 was significantly (*P* < 0.0001) affected by age and displayed a cubic trend (*P* < 0.0001) over time. The greatest relative expression of duodenal IL-10 was observed on days 3 and 12, both followed by a linear decrease (Figure 3A). Broilers sampled on day 21 post-hatch had the lowest duodenal IL-10 abundance. Contrastingly, ileal IL-10 relative abundance was lowest on day 3, then increased linearly until day 9, remained constant from day 9 to 15, followed by a decrease on days 18 and 21 (Figure 3C). Expression of IL-17 was greater on day 3 for both intestinal tissues. Relative abundance was lowest in the duodenum on day 18 (Figure 3C) and in the ileum on days 18 and 21 (Figure 3D). In a similar study, Song et al. (50) evaluated the development and functionality of the immune system in broilers from days 0 to 35 post-hatch by measuring relative amounts of immune-related genes in serum and ileum mucosa samples. This research group concluded that the immune system matures until days 30 to 34 and has not developed or functions well in 6- to 13-day-old broilers. Contrasting with our results, they observed lower transcription of cytokines in ileum of 7-day-old compared with 21-day-old broilers. Differences in sampling locations, target proteins, and quantification techniques may explain some of these differences.

### Intestinal Tight Junction Abundance Over Time

**Figure 4:**
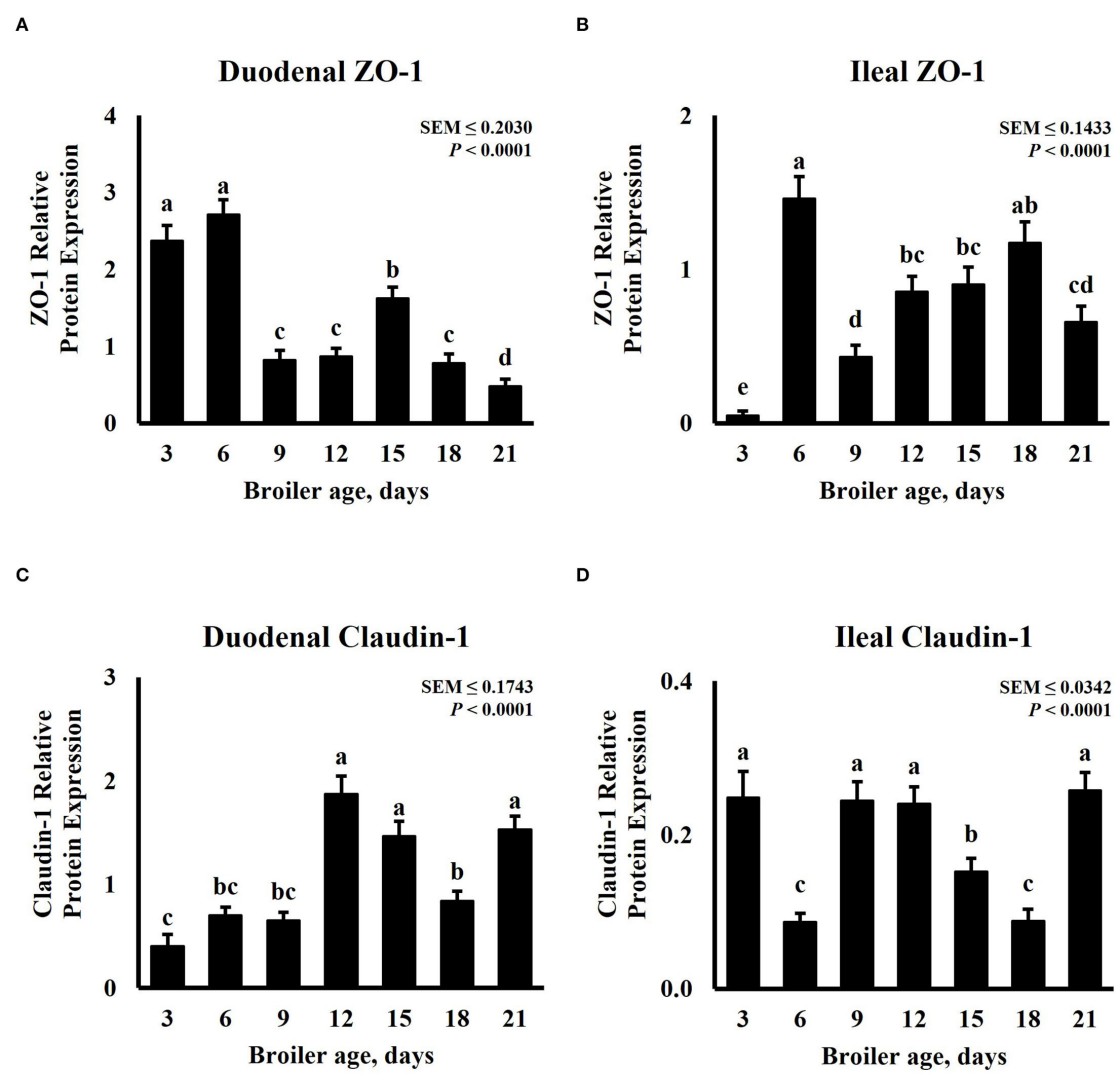
Effect of bird age on intestinal tight junction protein abundance. On each sampling day, a total of 24 birds (n = 12 birds per treatment) were selected for tissue collection. Protein expression was measured using quantitative, fluorescent Western Blot relative to total protein. **(A)** Duodenal ZO-1 abundance, **(B)** Duodenal Claudin-1 abundance, **(C)** Ileal ZO-1 abundance, and **(D)** Ileal Claudin-1 abundance. ^a−*e*^Bars with different superscripts differ at P ≤ 0.05.

In increased intestinal barrier integrity, tight junction expression is expected to increase. Tight junctions have a highly dynamic nature, these proteins can be remodeled and redistributed in response to various stimuli in different regions of the small intestine including dietary protein, amino acid concentration, and nutrient transporters (51). Intestinal tight junction protein abundance for this experiment is presented in Figure 4. The expression of ZO-1 and Claudin-1 was significantly (*P* < 0.0001) affected by age. The expression pattern of ZO-1 follows a cubic trend (*P* < 0.0001) for duodenum and ileum tissue samples, while that of Claudin-1 displays a quadratic trend (*P* ≤ 0.0401). Duodenal ZO-1 abundance was highest on days 3 and 6, proceeded by day 15, and was lowest in 21-day-old broilers (Figure 4A). However, ZO-1 abundance in the ileum was the lowest on day 3, increased on day 6, decreased on day 9, and linearly increased until day 18 (Figure 4B). In the duodenum, 12-, 15-, and 21-day-old broilers presented the greatest Claudin-1 relative expression, numerically lower abundance was observed on day 3 but was similar to days 6 and 9 (Figure 4C). Ileal Claudin-1 relative expression was the highest on days 3, 9, 12, and 21, the lowest on days 6 and 18, and intermediate on day 15 (Figure 4D). Claudin-1 results from this experiment differ from a similar experiment that measured mRNA levels of Claudin-1 and ZO-1 in the jejunum and ileum of broilers from days 0 to 14. In their experiment, Proszkowiec-Weglarz et al. (52) observed a constant decrease in gene expression of Claudin-1 from days 3 to 14 in both jejunum and ileum tissue samples. However, an increase in ZO-1 mRNA abundance was reported from days 3 to 10, followed by a decrease until day 14. Similar fluctuation in ZO-1 abundance in the ileum was observed in our experiment for different timepoints. It is important to note that Proszkowiec-Weglarz et al. (52) observed a higher expression of tight junction-related genes in both tissues 4 h post-hatch compared with the subsequent sampling time points. Functional maturation of GIT is triggered by microbiota and dietary antigens immediately after hatch (53). As bacterial communities in the intestine fluctuate along the life cycle of a chicken (54, 55), immune and tight junction proteins could be responding to these changes.

Our findings demonstrate cytokine downregulation by dietary _25_OHD_3_ supplementation in the small intestine of broiler chickens in an environment set for homeostatic conditions. Dietary _25_OHD_3_ supplementation decreased the expression of both anti- and pro-inflammatory molecules compared with broilers fed vitamin D_3_ as the only source of vitamin D. Distinct from previous work on poultry, where _25_OHD_3_ supplementation displayed regulation of cytokine genes only after the infliction of an immune challenge (21, 28, 29), this study suggests cytokine modulation in the ileum by dietary _25_OHD_3_ supplementation. Effects of _25_OHD_3_ supplementation on immune parameters were observed in the older birds, which could indicate the importance of this metabolite at later phases of growth. Therefore, future studies aimed at determining the effects of dietary _25_OHD_3_ inclusion on the intestinal abundance of immunomodulatory molecules should consider bird age and potential external stimuli.

## Data Availability Statement

The original contributions presented in the study are included in the article/Supplementary Material, further inquiries can be directed to the corresponding author.

## Ethics Statement

The animal study was reviewed and approved by the Auburn University Institutional Animal Care and Use Committee.

## Author Contributions

SL, LA, GA-P, JF, CS, and JS conducted the experiments, analyzed the samples, and collected the data. GA-P, JS, and CS analyzed the data. GA-P wrote the original manuscript draft. JS and CS oversaw all the experiments and revised the manuscript. All the authors contributed to the article and approved the submitted version.

## Funding

The study was supported by the United States Department of Agriculture National Institute of Food and Agriculture (USDA-NIFA) through Hatch Act funds to the Alabama Agricultural Experiment Station.

## Conflict of Interest

The authors declare that the research was conducted in the absence of any commercial or financial relationships that could be construed as a potential conflict of interest.

## Publisher's Note

All claims expressed in this article are solely those of the authors and do not necessarily represent those of their affiliated organizations, or those of the publisher, the editors and the reviewers. Any product that may be evaluated in this article, or claim that may be made by its manufacturer, is not guaranteed or endorsed by the publisher.
