# Supplementary material for: Dietary 25-Hydroxyvitamin D3 Supplementation Modulates Intestinal Cytokines in Young Broiler Chickens
Source: Front Vet Sci. 2022 Jul 11;9:947276. doi: 10.3389/fvets.2022.947276 (PMC9309538; doi:10.3389/fvets.2022.947276)
Supplement: Supplementary file 1 [file Data_Sheet_1.PDF]

Abascal-Ponciano et al., 2022
Supplementary information

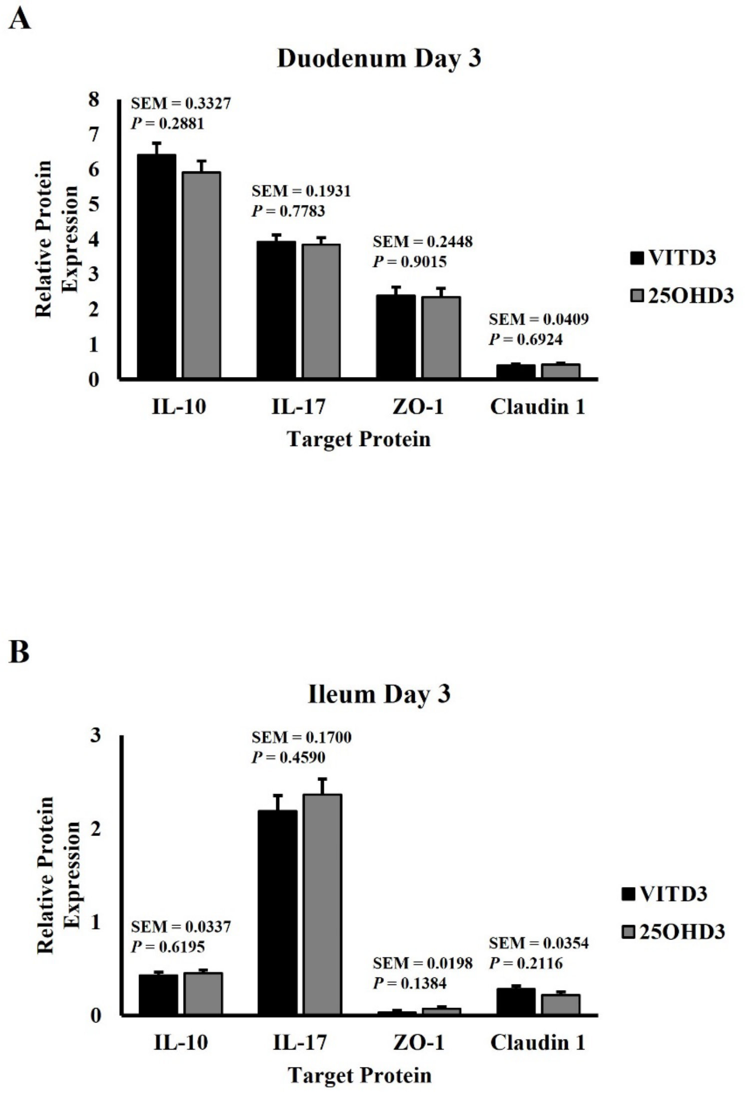

Supplementary Material Figure 1. Effect of dietary 25-hydroxycholecalciferol supplementation on 3-day-old broiler chicken intestinal protein abundance. Twenty-four birds were sampled on each sampling day (n = 12 birds of each treatment). Dietary treatments: VITD3 = 5,000 IU of vitamin D3 per kg of broiler chicken feed; 25OHD3 = 2,760 IU of 25-hydroxycholecalciferol + 2,240 of vitamin D3 per kg of broiler chicken feed. Protein expression was measured using quantitative, fluorescent Western Blot relative to total protein. **(A)** Duodenal IL-10, IL-17, ZO-1, and Claudin-1 protein abundance. **(B)** Ileal IL-10, IL-17, ZO-1, and Claudin-1 protein abundance.

A

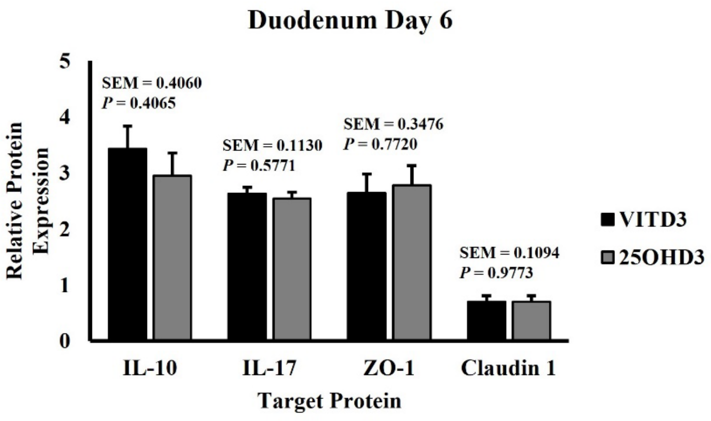

B

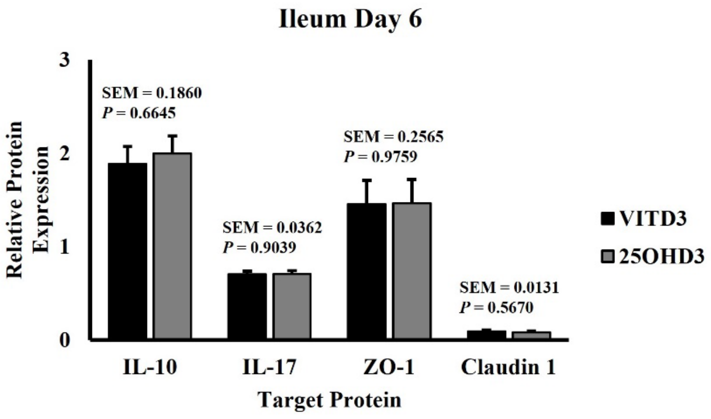

Supplementary Material Figure 2. Effect of dietary 25-hydroxycholecalciferol supplementation on 6-day-old broiler chicken intestinal protein abundance. Twenty-four birds were sampled on each sampling day (n = 12 birds of each treatment). Dietary treatments: VITD3 = 5,000 IU of vitamin D3 per kg of broiler chicken feed; 25OHD3 = 2,760 IU of 25-hydroxycholecalciferol + 2,240 of vitamin D3 per kg of broiler chicken feed. Protein expression was measured using quantitative, fluorescent Western Blot relative to total protein. **(A)** Duodenal IL-10, IL-17, ZO-1, and Claudin-1 protein abundance. **(B)** Ileal IL-10, IL-17, ZO-1, and Claudin-1 protein abundance.

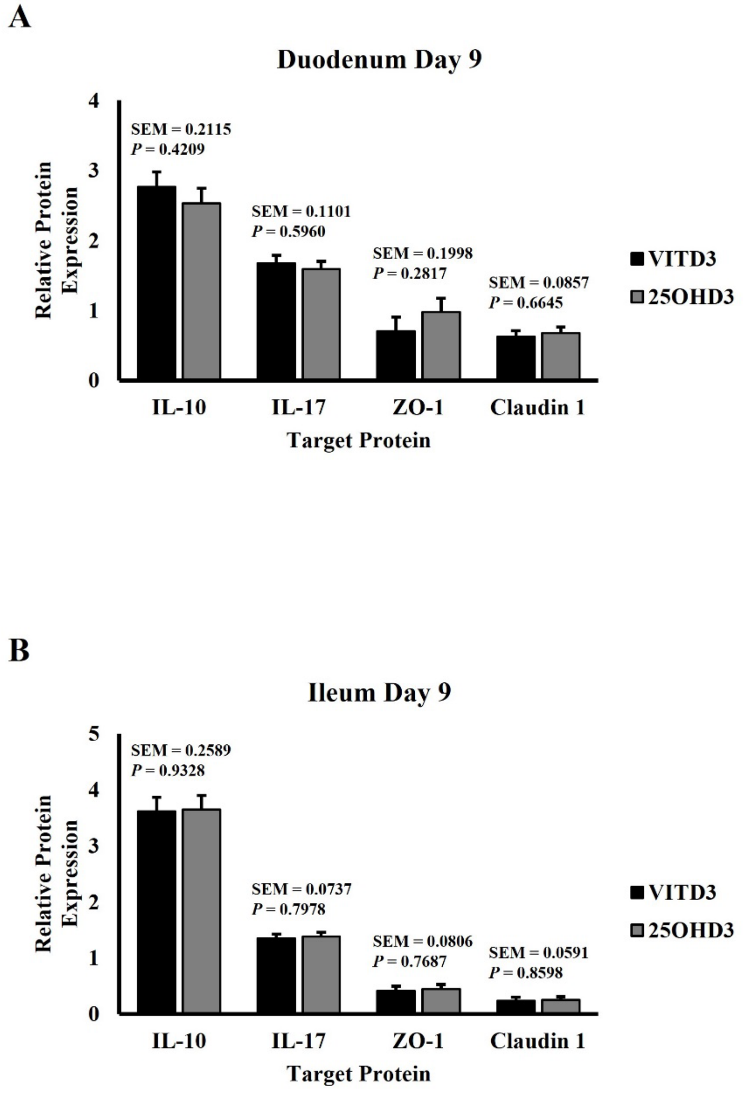

Supplementary Material Figure 3. Effect of dietary 25-hydroxycholecalciferol supplementation on 9-day-old broiler chicken intestinal protein abundance. Twenty-four birds were sampled on each sampling day (n = 12 birds of each treatment). Dietary treatments: VITD3 = 5,000 IU of vitamin D3 per kg of broiler chicken feed; 25OHD3 = 2,760 IU of 25-hydroxycholecalciferol + 2,240 of vitamin D3 per kg of broiler chicken feed. Protein expression was measured using quantitative, fluorescent Western Blot relative to total protein. **(A)** Duodenal IL-10, IL-17, ZO-1, and Claudin-1 protein abundance. **(B)** Ileal IL-10, IL-17, ZO-1, and Claudin-1 protein abundance.

A

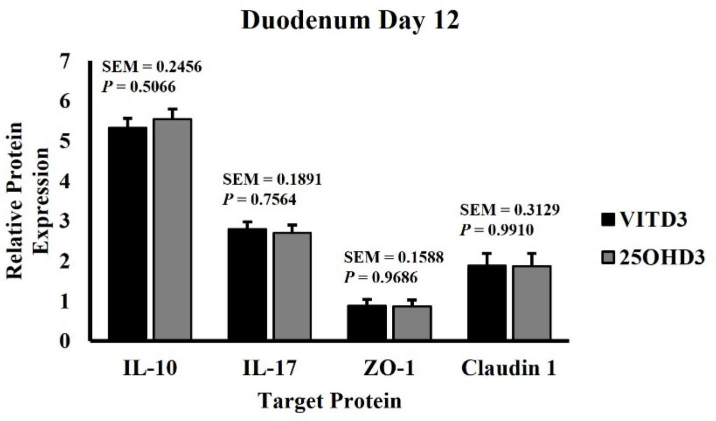

B

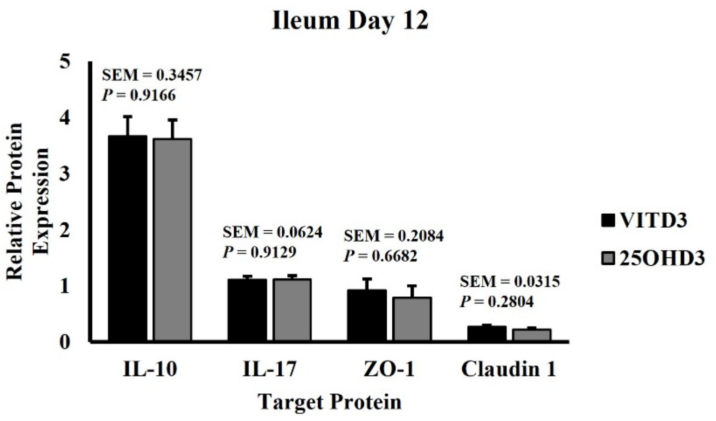

Supplementary Material Figure 4. Effect of dietary 25-hydroxycholecalciferol supplementation on 12-day-old broiler chicken intestinal protein abundance. Twenty-four birds were sampled on each sampling day (n = 12 birds of each treatment). Dietary treatments: VITD3 = 5,000 IU of vitamin D3 per kg of broiler chicken feed; 25OHD3 = 2,760 IU of 25-hydroxycholecalciferol + 2,240 of vitamin D3 per kg of broiler chicken feed. Protein expression was measured using quantitative, fluorescent Western Blot relative to total protein. **(A)** Duodenal IL-10, IL-17, ZO-1, and Claudin-1 protein abundance. **(B)** Ileal IL-10, IL-17, ZO-1, and Claudin-1 protein abundance.

A

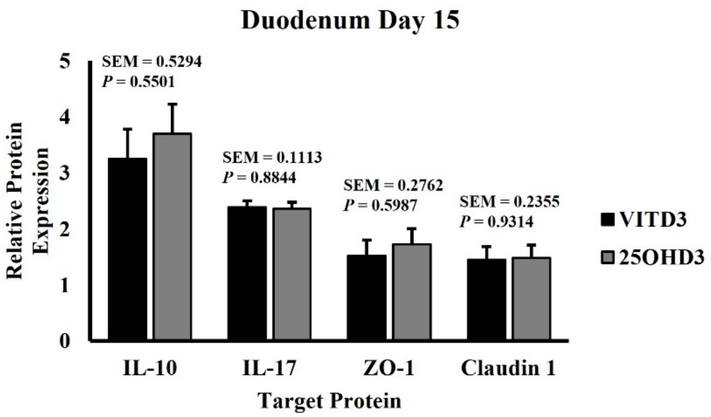

B

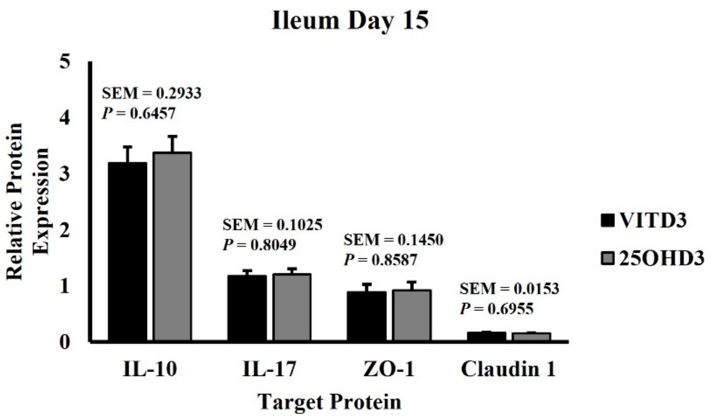

Supplementary Material Figure 5. Effect of dietary 25-hydroxycholecalciferol supplementation on 15-day-old broiler chicken intestinal protein abundance. Twenty-four birds were sampled on each sampling day (n = 12 birds of each treatment). Dietary treatments: VITD3 = 5,000 IU of vitamin D3 per kg of broiler chicken feed; 25OHD3 = 2,760 IU of 25-hydroxycholecalciferol + 2,240 of vitamin D3 per kg of broiler chicken feed. Protein expression was measured using quantitative, fluorescent Western Blot relative to total protein. **(A)** Duodenal IL-10, IL-17, ZO-1, and Claudin-1 protein abundance. **(B)** Ileal IL-10, IL-17, ZO-1, and Claudin-1 protein abundance.

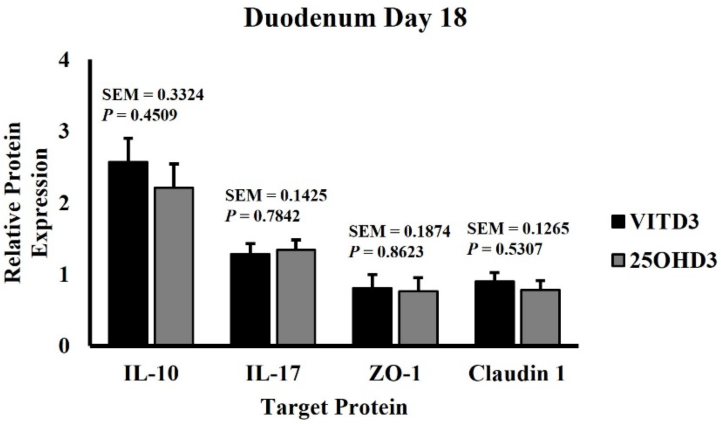

Supplementary Material Figure 6. Effect of dietary 25-hydroxycholecalciferol supplementation on 18-day-old broiler chicken duodenal IL-10, IL-17, ZO-1, and Claudin-1 protein abundance. Twenty-four birds were sampled on each sampling day (n = 12 birds of each treatment). Dietary treatments: VITD3 = 5,000 IU of vitamin D3 per kg of broiler chicken feed; 25OHD3 = 2,760 IU of 25-hydroxycholecalciferol + 2,240 of vitamin D3 per kg of broiler chicken feed. Protein expression was measured using quantitative, fluorescent Western Blot relative to total protein.

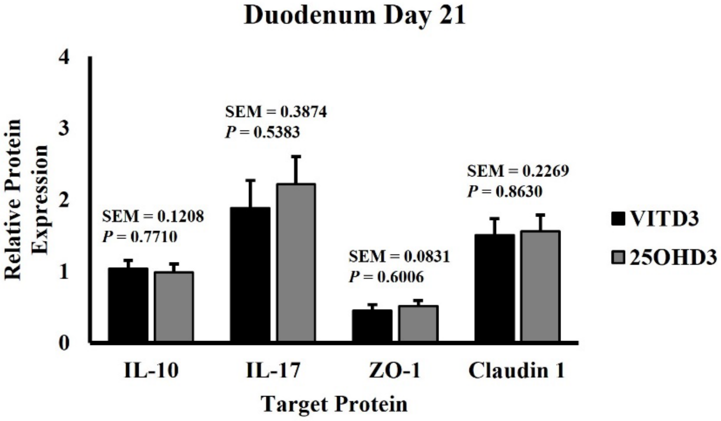

Supplementary Material Figure 7. Effect of dietary 25-hydroxycholecalciferol supplementation on 21-day-old broiler chicken duodenal IL-10, IL-17, ZO-1, and Claudin-1 protein abundance. Twenty-four birds were sampled on each sampling day (n = 12 birds of each treatment). Dietary treatments: VITD3 = 5,000 IU of vitamin D3 per kg of broiler chicken feed; 25OHD3 = 2,760 IU of 25-hydroxycholecalciferol + 2,240 of vitamin D3 per kg of broiler chicken feed. Protein expression was measured using quantitative, fluorescent Western Blot relative to total protein.
